# Multi-Parameter Auto-Tuning Algorithm for Mass Spectrometer Based on Improved Particle Swarm Optimization

**DOI:** 10.3390/bioengineering10091079

**Published:** 2023-09-12

**Authors:** Mingzheng Jia, Liang Li, Baolin Xiong, Le Feng, Wenbo Cheng, Wen-Fei Dong

**Affiliations:** 1 School of Biomedical Engineering (Suzhou), Division of Life Sciences and Medicine, University of Science and Technology of China, Hefei 230026, China; 2 Suzhou Institute of Biomedical Engineering and Technology, Chinese Academy of Sciences, Suzhou 215163, China; 3 Tianjin Key Laboratory of Medical Mass Spectrometry for Accurate Diagnosis, Tianjin 300399, China

**Keywords:** quadrupole mass spectrometer, auto-tuning algorithm, improved particle swarm optimization algorithm, inertia weights, dynamic boundaries

## Abstract

Quadrupole mass spectrometers (QMS) are widely used for clinical diagnosis and chemical analysis. To obtain the best experimental results, mass spectrometers must be calibrated to an ideal setting before use. However, tuning the current QMS is challenging. Traditional tuning techniques possess low automation levels and rely primarily on skilled engineers. Therefore, in this study, we propose an innovative auto-tuning algorithm for QMS based on the improved particle swarm optimization (PSO) algorithm to automatically find the optimal solution of QMS parameters and make the QMS reach the optimal state. The improved PSO algorithm is combined with simulated annealing, multiple inertia weights, dynamic boundaries, and other methods to prevent the traditional PSO algorithm from the issue of a local optimal solution and premature convergence. According to the characteristics of the mass spectrum peaks, a termination function is proposed to simplify the termination conditions of the PSO algorithm and further improve the automation level of the mass spectrometer. The results of auto-calibration testing of resolution and mass axis show that both resolution and mass axis calibration could effectively meet the requirements of mass spectrometry experiments. By the experiment of auto-optimization testing of lens and ion source parameters, these parameters were all in the vicinity of the optimal solution, which achieved the expected performance. Through numerous experiments, the reproducibility of the algorithm was established as meeting the auto-tuning function of the QMS. The proposed method can automatically tune the mass spectrometer from its non-optimal condition to the optimal one, which can effectively reduce the tuning difficulty of QMS.

## 1. Introduction

The quadrupole mass spectrometer (QMS) is a highly efficient mass spectrometer with excellent sensitivity and selectivity for various analytical applications [1,2], such as clinical diagnosis, the identification and quantification of biomolecules [3], drug metabolism studies [4], and environmental monitoring [5]. With the widespread use of QMS mass spectrometers in recent years, users not only inquire about the higher sensitivity of the instrument, but also pursue the intelligence and automation of the instrument, with analytical experiments performed quickly [6]. Mass spectrometer tuning is the process of optimizing the mass spectrometer parameters to achieve optimal experimental results before performing a sample analysis [7]. The goal of tuning is to obtain reasonable resolution and accurate mass axes, as well as acceptable peak intensities.

However, the mass spectral resolution and spectral peak intensity conflict with each other; that is, a higher resolution tends to degrade the intensity, while a higher intensity suppresses the resolution. Therefore, a trade-off between resolution and spectral peak intensity must be made in tuning and ensuring that both are met to obtain better experimental results. In addition, there is a degree of correlation between the resolution and mass axis, and the purpose of tuning is to find a balance between them such that the instrument can reach the optimal state.

Mass spectrometer tuning is divided into manual tuning and auto-tuning. In general, manual tuning is difficult and time consuming in addition to having inconsistent standards. Thus, auto-tuning is the current practice in mainstream instruments and is an important function in modern mass spectrometers, which can make mass spectrometry more rapid, accurate, and reliable, and help to improve the level of mass spectrometry automation. Currently, many studies focused on tuning mass spectrometry resolution in terms of automatic tuning of mass spectrometers. By adjusting the direct current (DC) and alternating current (AC) voltages applied to the quadrupole to change the resolution of the mass spectrometer, Kenny [8] proposed a method for adjusting the DC and AC voltages of the quadrupole to change the resolution of the mass spectrometer; however, the scheme design is not mentioned in this study. Syed et al. [9] proposed a ratiometric relationship between the DC and AC voltages and the resolution of the mass spectrometer, but did not realize the function of automatic resolution adjustment. Recently, Liu et al. [10] established an automatic mass resolution tuning algorithm, which equipped a corresponding model for tuning through the linear relationship between mass resolution and electrical parameters. However, the model only tuned the resolution without tuning the mass axis and spectral peak intensity. Moreover, the method often fails to achieve the optimal state of the instrument because of its use of iterative algorithms that fall into a state of local optimal solutions.

Therefore, how to solve the optimal solution problem in auto-tuning became a hot spot of concern. Automatic tuning methods based on heuristic optimization algorithms are more suitable for multi-parameter optimization, such as genetic algorithms, particle swarm optimization (PSO), and so on. The PSO algorithm is a heuristic optimization algorithm based on group intelligence, which has the advantages of simple implementation, fast convergence, and easy parallel computation, and is mostly used to solve combinatorial optimization, mode decomposition, sensor networks, biomolecular research, and other fields [11]. However, the traditional PSO algorithm has some limitations in optimization problems with multi-peak functions, such as poor convergence of the population of particles and poor global search ability of the algorithm [12,13,14,15,16]. To improve the performance of PSO algorithms, there were many studies on improved PSO algorithms. J. Riget introduced the attractive and repulsive PSO (ARPSO) in trying to overcome the problem of premature convergence, which uses a diversity measure to control the swarm [12]. Yu et al. proposed an enhanced PSO algorithm to enhance the convergence rate of iteration process [13]. Yu et al. proposed a nonparametric model for a magnetorheological elastomer-based isolator based on support vector regression (SVR), which employs a type of improved particle swarm optimization to optimize the parameters in SVR and has a better generalization capacity and better recognition accuracy than other conventional models [14]. F. Bergh proposed the cooperative particle swarm optimizer by using multiple swarms to optimize different components of the solution vector cooperatively to improve the performance of the traditional PSO algorithm [15]. Yue et al. proposed a PSO-BOA optimization strategy with applications in data classification [16]. The improvement of the traditional PSO algorithm is mainly divided into multiple populations to search; using topological structure to improve; combining with other evolutionary algorithms; improvement and change in iterative formula of velocity and position, etc., which enhance the convergence rate and search ability [15,16,17,18,19].

In this paper, we propose an improved particle swarm optimization (I-PSO) algorithm. This method introduces a simulated annealing (SA) algorithm, boundary dynamics, and improved multi-inertia weight selection, which improve the algorithm convergence rate and prevent the particles from falling into the local optimum. Additionally, we propose a QMS auto-tuning method based on an improved particle swarm optimization (I-PSO) algorithm. The I-PSO algorithm combined with simulated annealing and the multi-inertia weight algorithm is used to auto-tune the QMS instrument and optimize the quality of the spectra, which can make the QMS instrument reach the optimal state.

## 2. Methods

### 2.1. Standard PSO Algorithm and Simulated Annealing Algorithm

The PSO algorithm consists of M particles, each representing a potential solution. When the particles move, they consider their best position in the history and the group’s overall best position to gradually adjust their direction and speed toward the optimal solution [20].

The equation for particle *j* to adjust the velocity is shown in Equation (1).

(1) $$v_{j}(n+1)=wv_{j}(n)+c_{1}(p_{j}(n)-x_{j}(n))+c_{2}(g_{j}(n)-x_{j}(n)),j=1\cdots M$$

Here, w is the inertia weight, and constants $c_{1}$ and $c_{2}$ are random numbers, $p_{j}(n)$ is the historical best position of particle *j*, and $g_{j}(n)$ is the global best position of all particles. Equation (2) expresses the position-updating process for particle *j*.

(2) $$x_{j}(n+1)=x_{j}(n)+v_{j}(n+1),j=1\cdots M$$

Here, $x_{j}(n+1)$ and $v_{j}(n+1)$ represent the position coordinates and velocity vector, respectively, at the time step n+1.

The steps of the standard PSO algorithm [21] are as follows:

**Input:** Velocity boundary $v_{\min},v_{\max}$, position boundary $x_{\min},x_{\max}$,maximum number of iterations N, and initial velocity is set to 0.

**Step 1:** Initialization: Randomly generate the positions of all particles $x_{1}(0)\cdots x_{M}(0)$, and obtain the global best position g(0) at the current time. Proceed to Step 2.

**Step 2:** Calculate the velocity of each particle $x_{j}(n)$ at $v_{j}(n+1)$ according to Equation (1). If $v_{j}^{}<v_{\min}$ then $v_{j}^{}=v_{\min}$; if $v_{j}^{}>v_{\max}$ then $v_{j}^{}=v_{\max}$.

**Step 3:** Update the $x_{j}(n)$ position of each particle according to Equation (2),

if $x_{j}(n+1)<x_{\min}$ then $x_{j}(n+1)=x_{\min}$,

if $x_{j}(n+1)>x_{\max}$ then $x_{j}(n+1)=x_{\max}$. Go to Step 4.

**Step 4:** Calculating the global best position,

(3) $$g(n+1)=\arg\max\{f(x_{j}(i+1)),j=1\cdots M,i=0\cdots n\}$$

**Step 5:** Then check if the termination condition is met. If yes, terminate the algorithm. Otherwise, return to Step 2.

**Output:** Global best position g(n).

The simulated annealing (SA) algorithm is a versatile global optimization algorithm that works by exploring the solution space through random walks to find the best possible solution [22,23]. Two main processes are involved in the SA algorithm: the Metropolis algorithm and annealing process. The Metropolis algorithm is a randomized method that determines whether to accept a new solution as the current solution to prevent it from becoming stuck in a local optimal solution. The Metropolis algorithm criteria are expressed by Equation (4).

(4) $$P_{ij}=\left\{\begin{array}{c} 1\\ \exp[\frac{f(i)-f(j)}{T}] \end{array}\begin{array}{c} f(i)\geq f(j)\\ f(i)<f(j) \end{array}\right.$$

Here, *T* is the temperature parameter and f(i) and f(j) are the internal energies of the solid in states *i* and *j*, respectively.

When f(i)≥f(j), the SA algorithm accepts this new state with 100% probability. Therefore, the SA method eventually converges to the global optimal solution [24].

### 2.2. Improved PSO Algorithm

The standard PSO algorithm uses a constant inertial weight. If the inertia weight is low, the algorithm can easily determine the global optimal solution within the initial search space. However, if the optimal solution is outside this space, it may not be found. Nevertheless, if the inertia weight is large, the algorithm behaves more as a global search method and always searches for new areas. However, more iterations are required to reach the global optimal solution. If the inertia weight is moderate, the algorithm has a higher probability of finding the global optimal solution, but requires more iterations than in the first case. To address these issues, researchers proposed a method for random inertia weights. However, this method has high randomness [25,26,27]. In this study, a multi-inertia weight selection method was proposed based on the random inertia weight method. Thus, several inertia weights were added, several speeds were updated in each iteration, and several new positions were obtained. The best model was selected for comparison.

This was achieved by transforming the constant inertia weights in Equation (1) into a number of inertia weights that vary with the number of iterations. In each iteration, the inertia weights traverse all the elements in the set of inertia weights and find the new velocity corresponding to each element in the set of inertia weights. The set of inertia weights in this study contained four elements that linear weight, exponential weight, power weight and random weight. The mathematical form of the inertia weights are as follows [27,28,29].

Linear weight(L):

(5) $$w(n)=w_{\max}-\frac{n}{N}(w_{\max}-w_{\min})$$

Here, $w_{\max}$ is the max value of the weight, $w_{\min}$ is the min value of the weight, *N* is the iteration max value, *n* is the algorithm iterations.

Exponential weight (E):

(6) $$w(n)=w_{\max}e^{-\frac{n}{N}}$$

Here, $w_{\max}$ is the max value of the weight, *N* is the iteration max value, *n* is the algorithm iterations.

Power weight (P):

(7) $$w(n)=w_{\max}-(w_{\max}-w_{\min}){\left(\frac{n}{N}\right)}^{2}$$

Here, $w_{\max}$ is the max value of the weight, $w_{\min}$ is the min value of the weight, *N* is the iteration max value, *n* is the algorithm iterations.

Random weight(R):

(8) $$w(n)=rand(w_{\min},w_{\max})$$

Here, $w_{\max}$ is the max value of the weight, $w_{\min}$ is the min value of the weight, *n* is the algorithm iterations.

Another drawback of the standard PSO algorithm is its slow convergence rate, especially in the vicinity of the optimal solution; studies showed that the PSO algorithm tends to enter a stagnant state in the late evolutionary stage [30]. To resolve this problem, a dynamic boundary correction method can be added to the standard PSO algorithm; that is, the position boundary changes with the number of iterations. The advantage of the proposed method is that it improves the operational efficiency of the algorithm by weakening the oscillation amplitude when the algorithm converges, making it easier to satisfy the termination condition when the algorithm evolves to a late stage [31].

The dynamic correction of its boundary is determined by changing the fixed position boundary into a dynamic position boundary in Step 2 of the standard PSO algorithm. In general, the range of the dynamic position boundary is subsequently narrowed for faster convergence. The dynamic update of the position boundaries is calculated using Equations (9) and (10).

(9) $$x_{\min}=\left\{\begin{array}{cc} p_{j}(n)-\alpha \left|p_{j}(n)-x_{j}(n)\right| & p_{j}(n)\neq x_{j}(n)\\ p_{j}(n)-\beta \left|p_{j}(n)\right| & p_{j}(n)=x_{j}(n) \end{array}\right.$$

(10) $$x_{\max}=\left\{\begin{array}{cc} p_{j}(n)+\alpha \left|p_{j}(n)-x_{j}(n)\right| & p_{j}(n)\neq x_{j}(n)\\ p_{j}(n)+\beta \left|p_{j}(n)\right| & p_{j}(n)=x_{j}(n) \end{array}\right.$$

The probability of the particle jumping out of the local optimal solution is increased by enhancing the search capability of the algorithm by adding SA to the above two improvements. The steps of the improved PSO (I-PSO) algorithm with the addition of SA, boundary dynamics, and multiple inertial weights are as follows:

**Input:** Velocity boundary $v_{\min},v_{\max}$, position boundary $x_{\min},x_{\max}$, maximum number of iterations N, initial velocity is set to 0, parameter α,β, SA parameters ε, η, and T.

**Step 1:** Initialize: Randomly generate the positions of all the particles $x_{1}(0)\cdots x_{M}(0)$ and obtain the global best position g(0) at the current time. Proceed to Step 2.

**Step 2**: Update the position boundary $x_{\min},x_{\max}$ dynamically according to dynamic boundary Equations (9) and (10).

**Step 3**: The velocity $v_{j}(n+1)$ is calculated for each particle $x_{j}(n)$, as shown in Equation (7); however, four weights need to be calculated separately.

(11) $$v_{j}(n+1)=wv_{j}(n)+c_{1}(p_{j}(n)-x_{j}(n))+c_{2}(g(n)-x_{j}(n)),j=1\cdots M$$

Here, $w=w_{\Phi }(n),\Phi =\{L,E,P,R\}$ represents the four corresponding inertia weight Equations (5), (6), (7) and (8), respectively. For convenience, the four calculated velocities are denoted as $v_{j}^{L},v_{j}^{E},v_{j}^{P},v_{j}^{R}$.

If $v_{j}^{\Phi }<v_{\min}$ then $v_{j}^{\Phi }=v_{\min}$, else If $v_{j}^{\Phi }>v_{\max}$ then $v_{j}^{\Phi }=v_{\max}$.

**Step 4**: Update the position of each particle $x_{j}(n)$, as shown in Equation (2). According to the four velocities obtained in Step 3, four new positions, denoted as $x_{j}^{L}(n+1),x_{j}^{E}(n+1),x_{j}^{P}(n+1),x_{j}^{R}(n+1)$, are obtained. Subsequently, the optimal solutions for the four new positions were calculated.

(12) $$x_{j}(n+1)=\arg\max\{f(x_{j}^{\Phi }(n+1)),\Phi =\{L,E,P,R\}\}$$

If $x_{j}(n+1)<x_{\min}$ then $x_{j}(n+1)=x_{\min}$, else if $x_{j}(n+1)>x_{\max}$ then $x_{j}(n+1)=x_{\max}$.

Go to Step 5.

**Step 5**: Calculate the global optimal position and particle history optimal position, which are updated using the SA algorithm.

$$\Delta E_{j}=f(p_{j}(n))-f(x_{j}(n+1))$$

(13) $$\Delta E=f(g(n))-\max\{f(x_{j}(n+1)),j=1\cdots M\}$$

Based on this there are:

(14) pj(n+1)=argmax{f(xj(i+1)),i=0⋯n}e−ΔET>εfj(xj(n+1))e−ΔET≤ε

(15) g(n+1)=argmax{f(xj(i+1)),j=1⋯M,i=0⋯n}e−ΔET>εargmax{f(xj(n+1)),j=1⋯M}e−ΔET≤ε

T=ηT,η<1

Here, ε is a random number generated in a uniform distribution of [0, 1].

If the termination condition is reached, reach the end; otherwise, return to **Step 2**.

**Output**: Global best position g(n).

### 2.3. Auto-Tuning Algorithm for QMS Based on Improved PSO Algorithm

**Figure 1:**
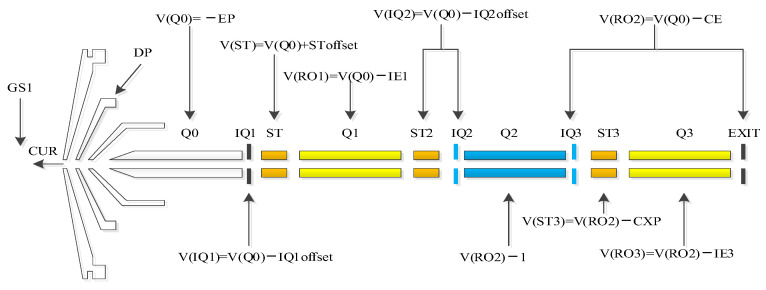
Schematic diagram of the lens and ion source parameters of a triple quadrupole mass spectrometer.

The tuning of QMS aims to make the mass spectrometer work optimally, which requires mass axis parameter calibration, resolution parameter (also known as the full width at half maximum (FWHM)) adjustment, and optimization of lens and ion source parameters for the mass spectrometer. The most commonly used QMS is the triple QMS, the ion optical schematic of which is shown in Figure 1.

**Figure 2:**
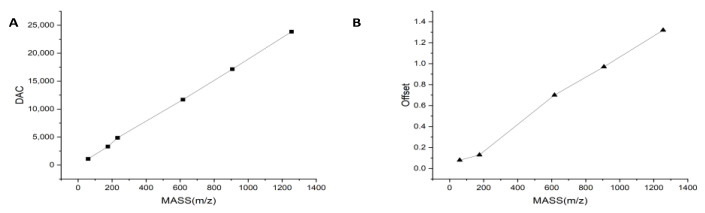
Correspondence of mass to DAC and mass to offset; (**A**) correspondence of mass to DAC; and (**B**) correspondence of mass to offset.

The mass axis of a QMS is determined by the linear proportionality between several mass numbers and digital-to-analog converters (DACs). For convenience, we formed pairs of mass numbers, DACs, and defined them as (MASS_1_,DAC_1_).... (MASS_n_,DAC_n_). The MAS–DAC curve can be adjusted by appropriately adjusting the DAC values. For example, if DAC3 is adjusted, the linear relationships between (MASS_2_,DAC_2_) and (MASS_3_,DAC_3_), (MASS_3_,DAC_3_) and (MASS_4_,DAC_4_) change. Similarly, the resolution is controlled by several MASS-OFFSET pairs, and the relative MASS-OFFSET curves can be changed by appropriately adjusting the corresponding offset values. The correspondence between the mass and DAC and the correspondence between the mass and offset are shown in Figure 2.

The lens parameters are a series of DC voltages acting on charged particles to obtain more ions from the mass analyzer and detector. To achieve the highest transfer efficiency, ion source parameters, which determine the ionization efficiency of the ion source, are also important for mass spectrometry. With proper settings of the lens and ion source parameters, the efficiency of material ionization and transport to the detector is significantly increased, and a better response can be obtained. Therefore, the optimization of these two types of parameters is crucial.

Based on the aforementioned characteristics of quadrupole mass spectrometry, we propose a termination condition evaluation function applicable to the improved PSO algorithm, as shown in Equation (16).

(16) $$f(x)=\frac{\lambda_{1}}{I}+\lambda_{2}{\left\|FW-TFW\right\|}^{2}+\lambda_{3}{\left\|MASS-TMASS\right\|}^{2},x={\left(x_{f},x_{len},x_{m}\right)}^{T}$$

Here, $x_{f}$, $x_{len}$, and $x_{m}$ are the resolution parameter vector, lens and ion source parameter vector, and mass axis parameter vector, respectively; I is the mass spectral peak intensity; FW and TFW are the current mass spectral peak FWHM and target FWHM of the mass spectral peak, respectively; MASS and TMASS are the detected mass numbers of the current mass spectral peak and target mass number of the mass spectral peak, respectively; and $\lambda_{1}$, $\lambda_{2}$, and $\lambda_{3}$ are non-negative constants. This evaluation function is also an important basis for determining whether an instrument is in optimal condition. In general, without any a priori knowledge, $x_{s}=\arg\min f(x)$ is the optimal value for any input parameter x, and thus the instrument is in the best condition. For the condition with a priori knowledge, a threshold value ε is set. The instrument is in the best condition when f(x)≤ε and the parameter satisfying f(x)≤ε is the optimal parameter.

**Figure 3:**
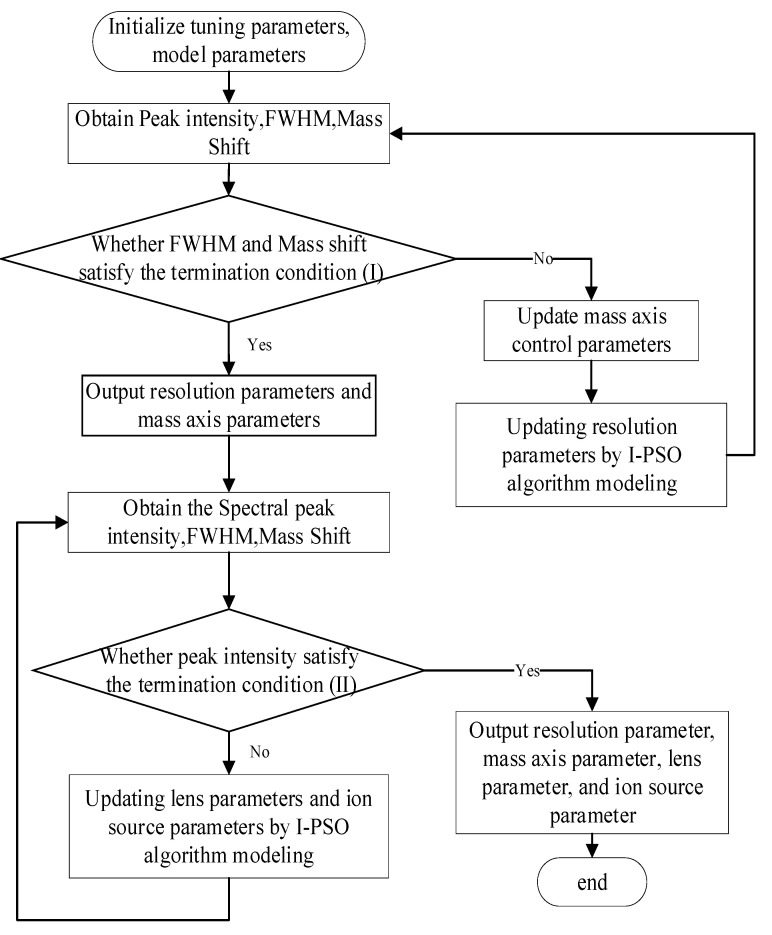
Flow chart of QMS auto-tuning algorithm based on improved PSO algorithm.

For a QMS, the tuning procedure encompasses three distinct stages: mass axis calibration, resolution calibration, and parameter optimization. The parameter optimization included lens parameter optimization and ion source parameter optimization. The initial steps involved calibration of the mass axis and resolution, and it is worth noting that these two aspects exhibited a limited correlation. During the execution, iterative alternation between the mass axis and resolution calibration was performed. Subsequently, the parameter optimization process commenced and concluded when the intensity reached a predefined threshold. At this point, the final values of the mass axis, resolution, and other parameters were obtained, thereby completing the tuning process. A flowchart of the QMS auto-tuning algorithm based on an improved PSO algorithm (I-PSO-Tune) is shown in Figure 3.

First, we discuss the mass axis calibration problem, where the mass axis control parameter shows a linear relationship (R > 0.99) with the mass number, which we assume to be of the form in Equation (17).

(17) DACs=ωMASS+ζ

Here, MASS is the mass number, DACs are the corresponding mass axis control parameter values, ω is the slope, and ζ is the intercept, which should be zero under ideal conditions. The correction equation for the mass axis is as in (18).

(18) DACs(n)=DACs(n−1)+ω(MASSd+MASS(n))

Here, DACs(n) is the DAC value obtained from the nth iteration, which is the DAC value of the last time of DACs(n − 1), ω is the slope in Equation (18), MASSd is the target mass number, that is, the mass number corresponding to the peak of the tuned liquid spectrum, and MASS(n) is the mass number corresponding to the peak of the nth detected spectrum.

Next, we discuss the resolution calibration problem. When adjusting the resolution of N feature mass numbers simultaneously with whose OFFSET value presents certain correlations, vectors comprising these N parameters are input to the improved PSO algorithm model with the output as the new OFFSET value. Termination conditions for the mass axis and resolution are shown in flowchart (I) as follows: set the parameters in Equation (12) to $\lambda_{1}=0$, $\lambda_{2}>0$, and $\lambda_{3}>0$.

Finally, we discuss the parameter optimization problem in which we set the termination condition (II) of the flowchart as follows: set the parameters in Equation (16) to $\lambda_{1}{=I}_{T}$, $\lambda_{2}=0$, and $\lambda_{3}=0$, where $I_{T}$ is the mass spectrum response value. Thus, the iterative stopping condition for parameter optimization is

(19) $$\begin{array}{c} \left\|f(x(n))\right\|\leq \varepsilon ,x(n)={\left(x_{f},x_{len},x_{m}\right)}^{T}(n)\;or\;n=N \end{array}.$$

Here, $\lambda_{1}{=I}_{T}$, $\lambda_{2}=0$ and $\lambda_{3}=0$, and ε=1.

## 3. Results and Discussion

### 3.1. Benchmark Function Test

In order to test the performance of the I-PSO algorithm, we select the standard PSO algorithm and I-PSO algorithm for a benchmark function test. In this, eight functions are selected to test the algorithm performance. All eight test functions in CEC2017 are evaluated as minimization problems and are classified as unimodal functions, simple multimodal functions, hybrid functions, and composite functions [32]. The test functions are shown in Table 1. The solution dimension of all test functions is 10, the population size is set to 100, and the search space is [−100, 100], and all algorithms are run independently on each test function 10 times, with the maximum number of iterations for each run being [100, 1500].

**Figure 4:**
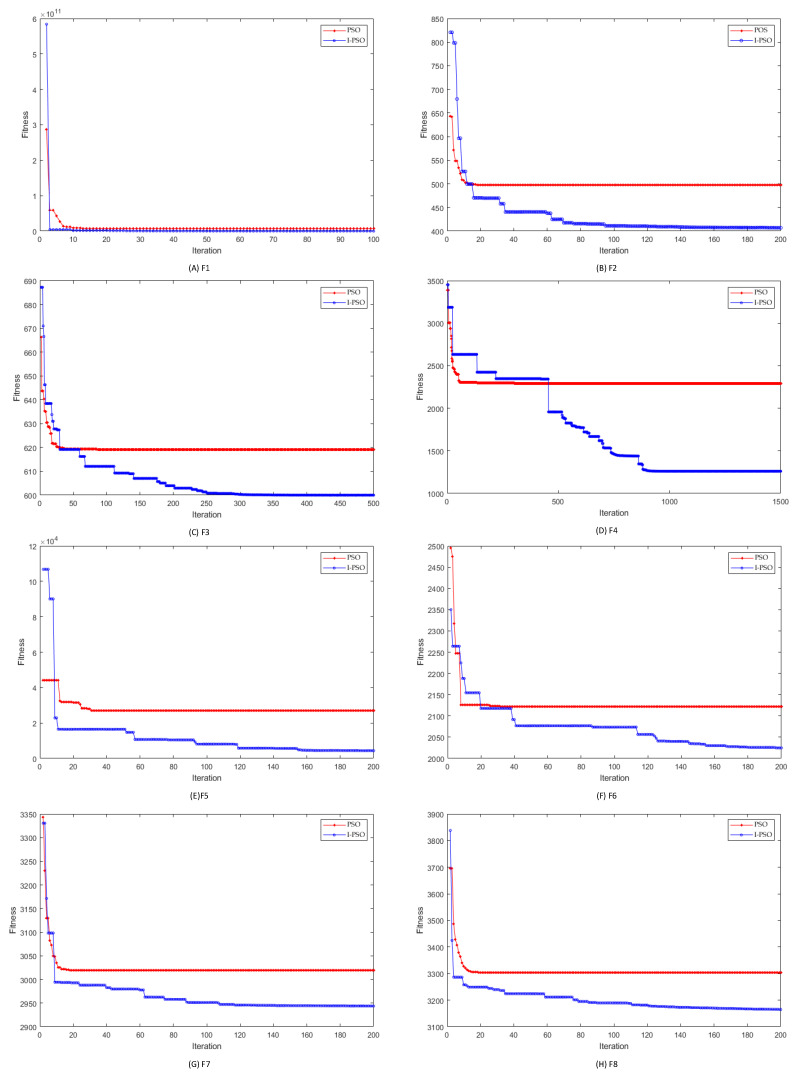
Comparison of the algorithm’s iterative performance on eight test functions.

Based on the above benchmark functions test results, it can be analyzed that I-PSO makes it easier to jump out of the local optimum and reach the global optimum point compared with the traditional PSO algorithm. The reason for this is that I-PSO adds the multi-inertia weight approach and the simulated annealing algorithm in PSO. The multi-inertia weight approach can expand the search range of the algorithm to be able to perceive more regions, while the SA algorithm provides the possibility of jumping out of the local optimum, because of the addition of simulated annealing, the I-PSO has a certain probability of rejecting the current optimum point, so as to achieve the purpose of jumping out of the local optimum solution. In the experiment, the function with multiple extreme points is selected, shaped as (E)F5~(H)F8 in Figure 4, and it can be seen that I-PSO can reach its optimal point; however, the traditional PSO will fall into the local optimum.

### 3.2. Auto-Tuning Performance Test

From the flowchart of the QMS auto-tuning algorithm, auto-tuning can be divided into two phases: calibration of the mass axis and resolution, and optimization of the lens and ion source parameters. Thus, in the following experiments, we used a triple QMS (Tianjin Guoke Medical Technology Development Co., Ltd., Tianjin, China), and the test sample was a polypropylene glycol PPG2000 solution with a concentration of 0.002 mol/mL and a relative molecular weight range of 20–1200. We tested these two stages, that is, the automatic calibration experiment of the mass axis and resolution, and the optimization experiment of the lens and ion source parameters, separately with correct and error-free results. The two processes were then combined and 10 tests were performed.

#### 3.2.1. Auto-Calibration Testing of Resolution and Mass Axis

To test optimization performance of the algorithm for the resolution and mass axis, we selected low, medium, and high mass numbers, and conducted resolution and mass axis tuning experiments simultaneously. Three mass numbers 59.05, 616.46, and 906.67 *m*/*z* were selected to represent low-, medium-, and high-mass segments, respectively.

The QMS requires an FWHM (unit–mass resolution) range of 0.5 to 0.7 *m*/*z* for low mass numbers, 0.6 to 0.8 *m*/*z* for medium mass numbers, and 0.6 to 0.85 *m*/*z* for high mass numbers. The mass accuracy of the full-mass segment should be no more than 0.2 *m*/*z*.

The following settings were selected as the input parameters: velocity boundary $v_{\min}=(-0.5,-60),v_{\max}=(0.5,60)$; parameter α=0.1,β=0.05; maximum number of iterations N=50; weight boundary $w_{\min}=(-8,0),w_{\max}=(8,65535)$; initial velocity is 0; and SA parameters ε=0.5, η=0.9, and T=80.

**Figure 5:**
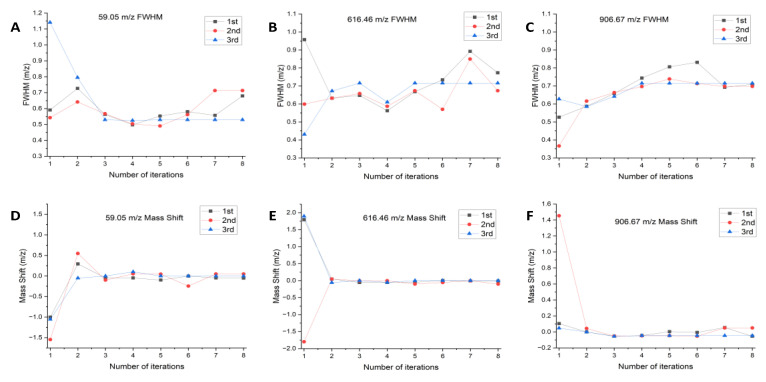
Iterative process for resolution calibration and mass axis. (**A**) 59.09 *m*/*z* iterative process for resolution calibration; (**B**) 616.46 *m*/*z* iterative process for resolution calibration; (**C**) 906.67 *m*/*z* iterative process for resolution calibration;(**D**) 59.09 *m*/*z* iterative process for mass axis; (**E**) 616.46 *m*/*z* iterative process for mass axis; (**F**) 906.67 *m*/*z* iterative process for mass axis.

The mass numbers were tested with values of 59.05, 616.46, and 906.67 *m*/*z*. The FWHM and mass axis of each mass number were obtained from three different initial positions, and calibration tests were performed until convergence to the required FWHM and mass accuracy range. The experimental results are shown in Figure 5.

After analyzing the data, the mass number 59.05 *m*/*z*, according to Figure 5A,D, three experiments of resolution calibration and mass axis reached the optimization at the fifth, third, and third iterations, the final output FWHMs were 0.5532, 0.5674, and 0.5297 *m*/*z*, and the corresponding mass shifts were −0.0523, −0.1000, and −0.0008 *m*/*z*; the mass number 616.46 *m*/*z*, according to Figure 5B,E, resolution calibration and mass axis of the three experiments were optimized at the eighth, fifth, and fifth iterations, and the final output FWHMs were 0.7399, 0.6738, and 0.7159 *m*/*z*, corresponding to mass shifts of −0.0133, −0.1025, and −0.0000 *m*/*z*; and the mass number 906.67 *m*/*z*, the three resolution calibration and mass axis experiments were optimized at the 8th, 4th, and 4th iterations, and the final output FWHM values were 0.7093, 0.6978, and 0.7149 *m*/*z*, corresponding to mass shifts of −0.0541, −0.0487, and −0.0463 *m*/*z*.

In the experimental procedure, we alternated between the resolution adjustment and mass axis calibration. Termination condition (I) was successfully satisfied, resulting in the final output values. The experiment demonstrated that both resolution and mass axis calibration could effectively meet the requirements of mass spectrometry experiments, regardless of the initial states (i.e., initial FWHM and mass axis). The difference observed in the number of iterations primarily corresponded to the spectral peaks where the FWHM and mass axis deviated significantly from the theoretical values. In such cases, a higher number of iterations is necessary to converge toward the theoretical values, which align with the fundamental principles of the algorithm.

#### 3.2.2. Auto-Optimization Testing of Lens and Ion Source Parameters

The optimization of the mass spectrometry lens and ion source parameters is based on the completion of the mass axis and resolution calibration, and we choose the output of one of the groups in Section 3.1 (mass axis parameters and resolution) as the initial conditions for the automatic optimization test of the lens and ion source parameters. In this test, we chose to optimize the parameters for low, medium, and high mass numbers and tested the mass numbers 59.05, 616.46, and 906.67 *m*/*z*, with a focus on the result of interest in the test being the spectral peak intensity. We set the passing intensity based on a priori knowledge, and during this intensity, the optimal parameters were considered to be achieved.

In this experiment, we chose the Q1POS scan mode of the mass spectrometer for testing, and four parameters were optimized; namely, curtain gas (CUR), atomization gas (GS1), declustering voltage (DP), and entrance voltage (EP).

In this paper, we form a vector of four parameters of the mass spectrometry lens and ion source parameters CUR, GS1, DP and EP, which we denote as (CUR,GS1,DP,EP), and these four parameters are tensored into a four-dimensional space. Corresponding to this four-dimensional space is Equation (12), which is in one-to-one correspondence. The following algorithm will perform iterative experiments on the vectors (CUR,GS1,DP,EP) of the four-dimensional space, expecting to obtain (CUR,GS1,DP,EP), which minimizes Equation (12).

**Figure 6:**
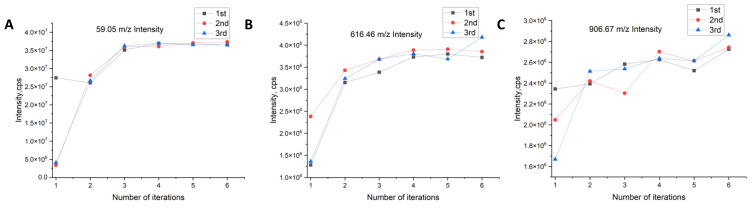
Iterative process for lens and ion source parameters. (**A**) 59.09 *m*/*z* iterative process for lens and ion source parameters; (**B**) 616.46 *m*/*z* iterative process for lens and ion source parameters; (**C**) 906.67 *m*/*z* iterative process for lens and ion source parameters.

The following settings were defined for the input parameters: velocity boundary $v_{\min}=(-5,-5,-1,-1),v_{\max}=(5,5,1,1)$, where $v_{\min},v_{\max}$ are the movement ranges of the vectors (CUR, GS1, DP, EP); parameters α=0.1,β=0.05, that determine the move step during the iteration of the vector (CUR,GS1,DP,EP); maximum number of iterations N=50; weight boundary $w_{\min}=(0,0,0,2),w_{\max}=(50,90,180,15)$,where $w_{\min},w_{\max}$ are the boundaries of the vector (CUR,GS1,DP,EP); initial velocity is 0; and SA parameters ε=0.5, η=0.9, and T=80. To test the performance of the lens and ion source parameter auto-tuning algorithm, the experimental design started with different initial parameters and was then tested for convergence to the required strength. The experimental results are shown in Figure 6.

After analysis, the Q1POS mode is optimized for four parameters, and the algorithm is terminated for an average of six iterations in three experiments for each quality number. The initial parameters of the three optimization tests for each quality number were different, and the outputs of the three experiments were the same after auto-optimization, and they were all in the vicinity of the optimal solution, which achieved the expected performance.

#### 3.2.3. Performance and Stability Testing

To verify the performance and stability of the algorithm, we executed the I-PSO-Tune auto-tuning algorithm several times and recorded experimental results. The experimental design started from the same initial state (mass axis and resolution parameters, lens, and ion source parameters) and was repeated 10 times; the results are shown in Table 2. The output results should satisfy the FWHM range of a low mass number: 0.5~0.7 *m*/*z*, the FWHM range of a medium mass number: 0.6~0.8 *m*/*z*, and the FWHM range of a high mass number: 0.6~0.85 *m*/*z*. Mass accuracy in the full-mass segment should be no more than 0.2 *m*/*z*.

As shown in Table 2, the auto-tuning algorithm for the QMS proposed in this study can meet 100% of the mass axis and resolution calibration requirements for mass numbers 59.05 and 906.67 *m*/*z*, and the calibration success rate of the mass axis and resolution for mass number 616.46 *m*/*z* is 90%, which suggests that the parameters of our algorithm need to be further optimized. In contrast, lens and ion source parameter optimization met the requirements, with coefficient of variation (CV) values of the 10 optimal intensities for mass numbers 59.05, 616.46, and 906.67 *m*/*z* as 5.5, 11.9, and 6.8%, respectively. The high CV value of 616.46 was due to one set of optimized intensities being low, which may be related to the state of the mass spectrum and peak identification at that time as well as to the set a priori threshold.

**Figure 7:**
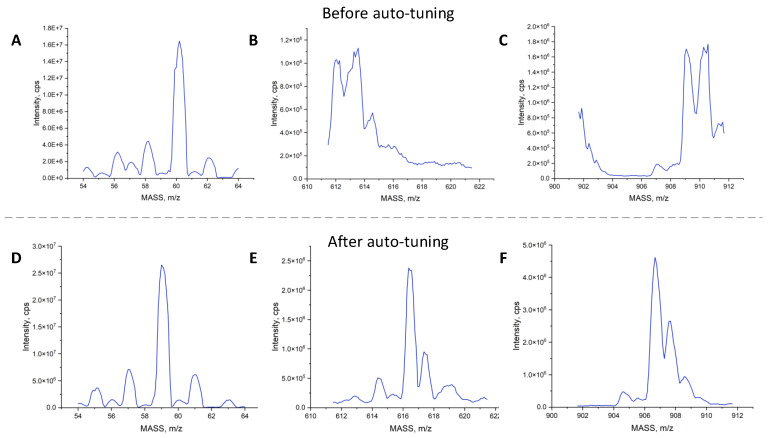
Spectra before auto-tuning and after auto-tuning by I-PSO-Tune algorithm. (**A**–**C**) 59.05 *m*/*z*, 616.46 *m*/*z* and 906.67 *m*/*z* spectra of before auto-tuning; (**D**–**F**) 59.05 *m*/*z*, 616.46 *m*/*z* and 906.67 *m*/*z* spectra of after auto-tuning.

Figure 7 shows the spectral signal from the mass spectrometer before and after auto-tuning. Before tuning, as shown in (A–C), the mass deviation was significant, the resolution of the spectral peaks was poor, and the peak intensity was low. However, the spectrum improved significantly after applying the auto-tuning algorithm proposed in this study, with better peak position, resolution, and intensity, as shown in (D–F). This result validates the effectiveness of the proposed algorithm in completing the instrument auto-tuning process.

In summary, the auto-tuning algorithm for the QMS proposed in this study was demonstrated to accomplish the instrument auto-tuning process. The proposed algorithm offers several advantages.

(1) This algorithm improves the intelligence level of the instrument, and the auto-tuning algorithm realizes the function of automatic optimization of the instrument compared with the manual tuning of the instrument, which still requires experienced engineers.
(2) This algorithm realizes that the traditional iterative algorithm can easily fall into the local optimal solution problem from a global perspective; thus, the instrument can be automatically tuned to the real optimal state.

## 4. Conclusions

Traditional mass spectrometer tuning relies on experienced engineers and is time consuming and inconsistent. Therefore, in this study, we propose an improved PSO auto-tuning algorithm based on the PSO algorithm by adding SA, boundary dynamics, and multiple inertia weights. The proposed algorithm solves the problem of the traditional PSO algorithm, which easily falls into a local optimal solution; thus, it is more suitable for quadrupole mass spectrometry auto-tuning. The experiments also showed that the algorithm could automatically tune an unoptimized mass spectrometer to the optimal state, and the repeatability of the algorithm was verified by repeating this experiment. The auto-tuning algorithm introduced in this study can be applied to the auto-tuning of a QMS.

In this study, the innovative auto-tuning algorithm based on the improved PSO algorithm is applied in QMS instruments to automatically adjust the parameters and make the QMS reach the optimal state. In the further work, we will try to apply this auto-tuning algorithm to other types of mass spectrometers, such as time-of-flight mass spectrometers, ion trap mass spectrometers, etc., to continuously improve the stability and versatility of the algorithm.

## Figures and Tables

**Table 1:** Test functions in CEC2017 [32].

Functions Type	Functions No.	Functions	$F_{i^{*}}=F_{i}(x^{*})$
Unimodal function	F1	Shifted and rotated sum of different power function $f_{2}(x)={\sum_{i=1}^{D}\left|x_{i}\right|}^{i+1}$	200
Simple multimodal functions	F2	Shifted and rotated rosenbrock’s function $f_{4}(x)=\sum_{i=1}^{D-1}\left(100{\left(x_{i}^{2}-x_{i+1}\right)}^{2}+{\left(x_{i}-1\right)}^{2}\right)$	400
	F3	Shifted and rotated expanded Scaffer’s F6 function $\begin{array}{l} f_{6}(x)=g(x_{1},x_{2})+g(x_{2},x_{3})+\cdots +g(x_{D-1},x_{D})+g(x_{D},x_{1})\\ g(x,y)=0.5+\frac{\left(\sin^{2}(\sqrt{x^{2}+y^{2}})-0.5\right)}{{\left(1+0.001(x^{2}+y^{2})\right)}^{2}} \end{array}$	600
	F4	Shifted and rotated Schwefel’s function $f_{10}(x)=418.9829\times D-\sum_{i=1}^{D}g(z_{i})$	1000
Hybrid functions	F5	Hybrid function 5 (N = 4) $F(x)=g_{1}(M_{1}z_{1})+g_{2}(M_{2}z_{2})+\cdots +g_{N}(M_{N}z_{N})+F^{*}(x),N=4$	1500
	F6	Hybrid function 6 (N = 6) $F(x)=g_{1}(M_{1}z_{1})+g_{2}(M_{2}z_{2})+\cdots +g_{N}(M_{N}z_{N})+F^{*}(x),N=6$	2000
Composition Functions	F7	Composition function 5 (N = 5) $F(x)=\sum_{i=1}^{N}\left\{\omega_{i}{}^{*}[\lambda_{i}g_{i}\left(x\right)+bias_{i}]\right\}+F^{*},N=5$	2500
	F8	Composition function 8 (N = 6) $F(x)=\sum_{i=1}^{N}\left\{\omega_{i}{}^{*}[\lambda_{i}g_{i}\left(x\right)+bias_{i}]\right\}+F^{*},N=6$	2800

**Table 2:** Auto-tuning repeatability experiment.

NO.	Mass Shift (*m*/*z*)			FWHM (*m*/*z*)			Intensity (CPS, MCA = 5)		
	59.05	616.46	906.67	59.05	616.46	906.67	59.05	616.46	906.67
1	−0.0981	0.0955	−0.0528	0.6537	0.6742	0.6569	3.33 × 10^7^	2.58 × 10^6^	2.63 × 10^6^
2	−0.0892	−0.1028	−0.0443	0.5950	0.6802	0.6900	3.13 × 10^7^	2.98 × 10^6^	2.64 × 10^6^
3	−0.0962	0.0463	−0.0952	0.6970	0.6170	0.6575	3.19 × 10^7^	1.93 × 10^6^	2.78 × 10^6^
4	−0.0500	0.0948	−0.1503	0.6259	0.6371	0.6170	3.26 × 10^7^	2.36 × 10^6^	2.22 × 10^6^
5	−0.0513	0.0942	−0.0447	0.6497	0.6298	0.6615	3.37 × 10^7^	2.37 × 10^6^	2.71 × 10^6^
6	−0.0489	0.0414	−0.1490	0.6239	0.7744	0.6063	3.32 × 10^7^	2.74 × 10^6^	2.47 × 10^6^
7	−0.0483	0.0983	−0.0495	0.6180	0.7929	0.6132	3.06 × 10^7^	2.77 × 10^6^	2.54 × 10^6^
8	0.0030	0.0419	−0.0510	0.5464	0.7758	0.6135	2.96 × 10^7^	2.89 × 10^6^	2.43 × 10^6^
9	−0.0010	0.1471	−0.0541	0.6519	0.6950	0.6408	2.89 × 10^7^	2.74 × 10^6^	2.62 × 10^6^
10	0.0003	0.2331	−0.0995	0.6340	0.6557	0.6177	2.96 × 10^7^	2.61 × 10^6^	2.33 × 10^6^

## Data Availability

Not applicable.
